# Anti-protozoal activity of aporphine and protoberberine alkaloids from *Annickia kummeriae* (Engl. & Diels) Setten & Maas (Annonaceae)

**DOI:** 10.1186/1472-6882-13-48

**Published:** 2013-02-27

**Authors:** Hamisi M Malebo, Tanja Wenzler, Monical Cal, Sauda M Swaleh, Maurice O Omolo, Ahmed Hassanali, Urs Séquin, Daniel Häussinger, Petur Dalsgaard, Matthias Hamburger, Reto Brun, Isaiah O Ndiege

**Affiliations:** 1 Department of Traditional Medicine Research, National Institute for Medical Research, P.O. Box 9653, Dar es Salaam, Tanzania; 2 Medical Parasitology and Infection Biology, Parasite Chemotherapy Unit, Swiss Tropical Institute, University of Basel, Socinstrasse 57, Basel, CH-4002, Switzerland; 3 Department of Chemistry, Kenyatta University, P.O. Box 43844, Nairobi, Kenya; 4 International Centre for Insect Physiology and Ecology, P.O. Box 30772, Nairobi, Kenya; 5 Institute of Organic Chemistry, University of Basel, St Johanns Ring 19, Basel, CH-4052, Switzerland; 6 Institute of Pharmaceutical Biology, University of Basel, Klingelbergstrasse 50, Basel, CH-4056, Switzerland; 7 Department of Pure and Applied Chemistry, Masinde Muliro University of Science & Technology, P. O. Box 190, Kakamega, Kenya

**Keywords:** *Annickia kummeriae*, *Enantia kummeriae*, Annonaceae, Alkaloids, Aporphine, Protoberberine, Antiplasmodial, Antitrypanosomal, Antileishmanial, Cytotoxicity

## Abstract

**Background:**

Malaria, trypanosomiasis and leishmaniasis have an overwhelming impact in the poorest countries in the world due to their prevalence, virulence and drug resistance ability. Currently, there is inadequate armory of drugs for the treatment of malaria, trypanosomiasis and leishmaniasis. This underscores the continuing need for the discovery and development of new anti-protozoal drugs. Consequently, there is an urgent need for research aimed at the discovery and development of new effective and safe anti-plasmodial, anti-trypanosomal and anti-leishmanial drugs.

**Methods:**

Bioassay-guided chromatographic fractionation was employed for the isolation and purification of antiprotozoal alkaloids.

**Results:**

The methanol extract from the leaves of *Annickia kummeriae* from Tanzania exhibited a strong anti-plasmodial activity against the multi-drug resistant *Plasmodium falciparum* K1 strain (IC_50_ 0.12 ± 0.01 μg/ml, selectivity index (SI) of 250, moderate activity against *Trypanosoma brucei rhodesiense* STIB 900 strain (IC_50_ 2.50 ± 0.19 μg/ml, SI 12) and mild activity against *Leishmania donovani* axenic MHOM-ET-67/82 strain (IC_50_ 9.25 ± 0.54 μg/ml, SI 3.2). Bioassay-guided chromatographic fractionation led to the isolation of four pure alkaloids, lysicamine (**1**), trivalvone (**2**), palmatine (**3**), jatrorrhizine (**4**) and two sets of mixtures of jatrorrhizine (**4**) with columbamine (**5**) and palmatine (**3**) with (−)-tetrahydropalmatine (**6**). The alkaloids showed low cytotoxicity activity (CC_50_ 30 - >90 μg/ml), strong to moderate anti-plasmodial activity (IC_50_ 0.08 ± 0.001 - 2.4 ± 0.642 μg/ml, SI 1.5-1,154), moderate to weak anti-trypanosomal (IC_50_ 2.80 ± 0.001 – 14.3 ± 0.001 μg/ml, SI 2.3-28.1) and anti-leishmanial activity IC_50_ 2.7 ± 0.001 – 20.4 ± 0.003 μg/ml, SI 1.7-15.6).

**Conclusion:**

The strong anti-plasmodial activity makes these alkaloids good lead structures for drug development programs.

## Background

Protozoal diseases such as malaria, trypanosomiasis and leishmaniasis have an overwhelming impact in the poorest countries in the world [1]. Due to their prevalence, virulence and drug resistance, they are the most serious and widespread parasitic diseases in the tropics [1-5]. The inadequate armory of drugs for the treatment of malaria, trypanosomiasis and leishmaniasis; and the high cost of new drugs coupled with the rapid development of resistance to new anti-parasitic drugs are some of the limiting factors in the fight against these tropical diseases. This underscores the continuing need for the discovery and development of new anti-protozoal drugs. Consequently, there is an urgent need for research aimed at the discovery and development of new effective and safe anti-plasmodial, anti-trypanosomal and anti-leishmanial drugs. In view of the complicated situations in dealing with parasitic infections, chemotherapy remains a dependable strategy in disease control. In the development of new drugs, the plant kingdom is considered to be important source for lead compounds owing to the successful use in traditional treatment of various ailments since antiquity [6]. Historically, medicinal plants have served as sources of new pharmaceutical products like quinine and artemisinin [7] and inexpensive starting materials for the synthesis of many known drugs. Research focused on the identification of medicinal natural products from higher plants for the discovery of new parasitic agents has been ongoing for more than five decades.

**Figure 1:**
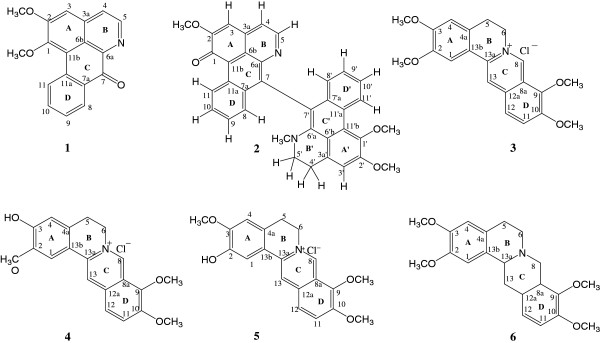
Chemical structures of isolated compounds.

Ethnomedical information revealed that several *Annickia* (formerly *Enantia)* species are used widely for the treatment of malaria and other ailments [8]. *Enantia chlorantha* and *E. polycarpa* are used traditionally in the treatment of malaria and fever in West and Central Africa [9,10]. Consequently, previous pharmacological investigations on genus *Enantia* revealed promising anti-protozoal activity with the stem-bark extract of *E. chlorantha* showing strong *in vitro* anti-plasmodial activity against *P. falciparum* K1 strain (IC_50_ 0.126 μg/ml) and good selectivity (SI 616) [11]. Furthermore, *E*. *chlorantha* aqueous and ethanolic extracts exhibited *in vivo* activity with ED_50_ values of 6.9 mg g-1 and 0.34 mg g-1, respectively, against *Plasmodium yoelii* in experimentally infected mice [12]. The chemistry of *E. chlorantha* and *E. polycarpa* has been extensively studied [10,13-15]. Several quinoline and isoquinoline alkaloids including protoberberines, quinine and dihydroquinidine have been isolated from *E. polycarpa*[16,17]. Protoberberine alkaloids have been identified as the major anti-protozoal alkaloids in *E. chlorantha* and *E. polycarpa*[16-19]. Protoberberines isolated from *Enantia chlorantha* exhibited significant antiplasmodial activity against both CQ-sensitive and resistant strains of *P. falciparum*: palmatine (**3**) (IC_50_ 0.27 and 0.16 μg/ml, respectively) and jatrorrhizine (**4**) (IC_50_ 4.2 and 1.61 μg/ml, respectively) *in vitro*[18]. A mixture of protoberberine alkaloids from *Enantia chlorantha* containing; palmatine (**3**), jatrorrhizine (**4**) and columbamine (**5**) (Hepasor), were shown to prevent liver injury from chemically induced traumatization and also promoted the healing process after the injury [20] in experimental mice. Palmatine (**3**) and jatrorrhizine (**4**) demonstrated to inhibit the growth of *Babesia gibsoni* at concentrations ranging from 100 and 10 μg/ml [21]. In an effort to identify the molecular basis of activity, we undertook bioassay-guided fractionation of extracts of *Annickia kummeriae* (Engl. & Diels) Setten & Maas (formerly, *Enantia kummeriae*), a plant traditionally used for the treatment of malaria in Tanzania. Bioassay-guided chromatography led to the isolation of lysicamine (**1**), trivalvone (**2**), palmatine (**3**), jatrorrhizine (**4**) and two sets of mixtures of jatrorrhizine (**4**) with columbamine (**5**) and palmatine (**3**) with (−)-tetrahydropalmatine (**6**) as shown in Figure 1.

## Methods

### General procedures

Analytical grade and double-distilled solvents were used for the extraction and chromatographic isolation and purification of compounds. Analytical thin layer chromatography (TLC) was performed on both aluminium and plastic sheets precoated with silica gel 60 F_254_ (Merck) with a 0.2 mm layer thickness. Visualisation of TLC spots was carried out under UV light at 254 or 366 nm and by spraying with Dragendorff reagent. Preparative thin layer chromatography (PTLC) was done using normal phase silica gel 60 F_254_ (Merck) precoated on glass plates (20 × 20 cm), with varying thickness (0.5, 1.0 or 2.0 mm). Detection was done under UV light at 254 or 366 nm. Preparative high speed counter-current chromatograph (HSCCC) was done on Potomac (P.C. Inc., Buffalo, NY-USA) equipped with three preparative multilayer coils (wound with 1.7 mm internal diameter, polytetrafluoroethylene PTFE tubing of 80 ml and 240 ml connected in series with a total capacity of 320 ml) run at a revolution speed of 611 rpm and the solvent was pumped into the column with a Büchi B-688 chromatography pump. Continuous monitoring of the effluent was achieved with a Model UV-II detector Monitor at 254 nm. A manual sample injection valve with a 20 mL loop was used to introduce the sample into the column and the eluent collected in a Büchi B-684 fraction collector. Melting points of recrystallized solids were measured on a Büchi B-540 apparatus and are uncorrected. IR spectra were measured on a Perkin Elmer model 1600 FT-IR spectrophotometer using potassium bromide pellets. Mass spectra were measured on mass spectrometer VG 70S (EIMS) and a Finnigan MAT 312 FABMS. NMR spectra were measured on Bruker Avance 400 (^1^H NMR 400 MHz; ^13^C NMR 101 MHz), Bruker VRX 500 (^1^H NMR 500 MHz; ^13^C NMR 125 MHz) and Bruker DRX 600 (^1^H NMR 600 MHz; ^13^C NMR 150.9 MHz). The purity level was determined by LC-MS (Agilent 1100 system equipped with an Agilent 1100 DAD MS detector; column Nucleodur C_18_, 5 μm, 125 mm × 4.0 mm internal diameter (i.d); mobile phase A: 0.01% aqueous formic acid and mobile phase B: acetonitrile). The structures were assigned by NMR and mass spectrometry. The isolated compounds were screened for anti-plasmodial, anti-trypanosomal, anti-leishmanial and cytotoxic activity.

### Plant materials and chemicals

Plant materials were collected at Amani Nature Reserve (Tanzania) in August 2003 and identified at the Department of Botany, University of Nairobi (Kenya) where the voucher specimen (HM 2004/04) is deposited in the Herbarium. The plant materials (leaves, root-bark and stem-bark) were dried under shade for 14 days and ground to powder. The ground air-dried *Annickia kummeriae* leaves, stem and root bark (1.12, 1.55 and 1.77 kg, respectively) were extracted sequentially, at room temperature for 48 hours with intermittent shaking, with petroleum ether (PE), dichloromethane (DCM) and methanol (MeOH). The extract was filtered off, the solvent removed under reduced pressure at 30°C, dried further under a stream of nitrogen for 24 hours before being weighed and used for biological assays.

Chemicals used were: Formic acid, hydrochloric acid, sulphuric acid, acetic acid, citric acid, *p*-anisaldehyde, vanillin, dragendorf reagent, sodium chloride, sodium hydrogen carbonate, acetone, *n*-hexane, petroleum ether, dichloromethane, chloroform, ethyl acetate, toluene, ethanol and methanol were also bought from Kobian Chemicals, Nairobi, Kenya and Fluka AG in Switzerland. Analytical grade or double-distilled solvents were used for the extraction and chromatographic isolation and purification of compounds. [^3^H]-Hypoxanthine and Rosewell Park Memorial Institute 1640 (RPMI 1640) powdered medium were bought from Gibco Laboratories, California, U.S.A whereas, dextrose, Giemsa stain, resazurin dye, glycerol and *N*-2-hydroxyethylpiperazine *N*-2-ethanesulfonic acid (HEPES) were bought from Sigma-Aldrich, Germany. Deuterated solvents: chloroform and methanol used for spectroscopic analysis were bought from Fluka AG, Switzerland.

### Bioassay of extracts and guided isolation of aporphine and protoberberine alkaloids

#### *In vitro* anti-plasmodial assay

Anti-plasmodial activity was evaluated against the multi-drug resistant *Plasmodium falciparum* K1 strain (resistant to chloroquine and pyrimethamine), using the parasite cultivation method of Trager and Jensen, 1976 [22] and the assay originally described by Desjardins *et al.,* 1979 [23] with slight modifications by Matile & Pink [24].

#### *In vitro* anti-trypanosomal assay

The *in vitro* anti-trypanosomal activity was evaluated against *Trypanosoma brucei rhodesiense* STIB 900 strain, using the cultivation method of Baltz *et al.,*1985 [25] whereby the Minimum Essential Medium (MEM) was supplemented with 0.2 mM 2-mercaptoethanol, 1 mM sodium pyruvate, 0.5 mM hypoxanthine and 15% heat-inactivated horse serum. The assay was performed according to Räz *et al.,* 1997 [26].

#### *In vitro* anti-leishmanial assay

The *in vitro* anti-leishmanial assay was carried out against axenic amastigote forms of *Leishmania donovani* MHOM-ET-67/82 strain according to the procedure described by Ganapaty *et al.,* 2006 [27].

### Cytotoxicity assay

The *in vitro* cytotoxicity assay was carried out using rat skeletal myoblast (L-6) cells according to the procedure described by Ganapaty *et al.,* 2006 [27]. Cytotoxicity activity of the test extract and compounds (IC_50_) was compared with cytotoxicity activity of the standard cytotoxic compound and used to calculate selectivity index. Selectivity indices (SI) were calculated using the formula:

$$\text{SI}=\frac{\text{Cytotoxicity}\;\text{of}\;\text{standard}\;\text{drug}\;\left({\text{CC}}_{50}\right)}{\text{Cytotoxicity}\;\text{of}\;\text{test}\;\text{extract}/\text{compound}\;\left({\text{IC}}_{50}\right)}$$

### Bioassay guided isolation of antiplasmodial compounds

The ground air-dried leaves, stem bark and root bark of *Annickia kummeriae* (1.12 kg, 1.55 kg and 1.77 kg, respectively) was extracted sequentially with solvents of increasing polarity (petroleum ether, dichloromethane and methanol) for 48 hours at room temperature. The resulting extracts were obtained by filtration and concentration *in vacuo* at 30°C. After screening for anti-plasmodial, anti-trypanosomal, anti-leishmanial and cytotoxic activity, the crude methanolic leaf extract, which was the most active, was selected for bioassay-guided fractionation and isolation of anti-protozoal compounds. The methanolic leaf extract (3 g) was fractionated using HSCCC through stepwise elution with a biphasic solvent system (CHCl_3_:MeOH:0.2 M HCl 7:3:4, v/v/v) to yield 17 fractions which were screened for anti-plasmodial and cytotoxic activity. The HSCCC fractions AKLM 4-AKLM 6 and AKLM 7-AKLM 10, which exhibited anti-plasmodial activity, were combined based on similarity of the TLC profile. Repeated HSCCC of fraction AKLM 2 using stepwise elution with a biphasic solvent system (CHCl_3_:MeOH:0.2 M HCl 7:3:4) gave 11 sub-fractions (AKLM 1-AKLM 11) which were screened for anti-plasmodial and cytotoxic activity. Column chromatography of sub-fractions AKLM 2.10 and AKLM 2.11 on silica gel (0.040–0.063 mm) eluting with C_6_H_14_-EtOAc 1:1, and EtOAc-MeOH 8:2 followed by purification with sephadex LH-20 eluting with MeOH-CHCl_3_ 1:1 and preparative TLC (PTLC) on silica gel PF_254_ with CHCl_3_:MeOH:HCO_2_H 98:1.8:0.2 yielded (10.21 mg) of lysicamine (**1**) ( 0.01% yield, 92% purity) and (8.10 mg) of trivalvone (**2**) (0.01% yield:, 95% purity), respectively. Repeated HSCCC eluting with CHCl_3_:MeOH:0.2 M HCl 7:3:4 of the combined fractions AKLM 7-AKLM 10 gave 20 sub-fractions (AKLM 7.1-AKLM 7.20). TLC analysis indicated a similar pure compound in AKLM 7.6-AKLM 7.13 which was recrystallized from methanol to yield (1.52 g) of palmatine (**3**) (1.84% yield, 91% purity). Column chromatography (silica gel 0.040–0.063 mm) of sub-fractions AKLM 7.15-AKLM 7.16, with similar TLC profiles, eluting sequentially with CHCl_3_:MeOH:HCO_2_H 9:0.75:0.25, 8:1.75:0.25, 6:3.75:0.5 and 5:4.5:0.5 followed by recrystallization from methanol yielded (40.82 mg) of jatrorrhizine (**4**) (0.05% yield, 94% purity). Repeated HSCCC of of the combined fractions AKLM 4-AKLM 6 with CHCl_3_:MeOH:0.2 M HCl 7:3:4 gave 16 sub-fractions (AKLM 4.1-AKLM 4.16). Column chromatography (silica gel 0.040–0.063 mm) with MeOH;CH_2_Cl_2_:HCO_2_H 4:15:1 followed by PTLC (silica gel PF_254_) with MeOH-CH_2_Cl_2_-HCO_2_H 5:14:1 gave (34.2) of an inseparable mixture (1.2:1.0) of jatrorrhizine (**4**) and columbamine (**5**) (0.04% yield). Similarly, HSCCC of of AKLM 16 with CHCl_3_:MeOH:0.2 M HCl 7:3:4 gave 12 sub-fractions (AKLM 16.1-AKLM 16.12). Column chromatography (silica gel 0.040–0.063 mm) of the combined sub-fractions AKLM 16.8-AKLM 16.10 with MeOH;CH_2_Cl_2_;HCO_2_H 5:14:1 followed by PTLC (silica gel PF_254_) with MeOH:CH_2_Cl_2_-HCO_2_H 4:15:1 yielded (28.2 mg) of an inseparable mixture (1.1:1.0) of palmatine (**3**) and (−)-tetrahydropalmatine (**6**) (0.03% yield).

### Structural elucidation of isolated compounds

The chemical structures of isolated compounds were established on the basis of spectroscopical data as Infra-red (IR), 1D (^1^H, ^13^C, DEPT 135) and 2D-NMR experiments; Heteronuclear Multiple-Quantum Correlation (HMQC), correlation spectroscopy (COSY) and Heteronuclear Multiple Bond Correlation (HMBC) plus Mass Spectroscopy (MS) data. The ^13^C NMR data were assigned with the help of HMQC and DEPT 135 experiments while, the connectivity’s of the molecular fragments were established by HMBC, COSY and NOESY. The analysis of the spectra and structure elucidation was also facilitated by comparison of observed and published ^1^H and ^13^C NMR data for the compounds.

Lysicamine (**1**): yellow needles (10.21 mg), m.p. 209–211°C, ^1^H NMR (CDCl_3_, 600 MHz) δ 7.57 (1H, s, H-3), 8.07 (1H, d*, J* = 5.2 Hz, H-4), 8.77 (1H, d*, J* = 5.2 Hz, H-5), 8.48 (1H, dd*, J* = 9.0, 1.8 Hz, H-8), 7.63 (1H, t*, J* = 9.0, 1.2 Hz, H-9), 7.86 (1H, t*, J* = 9.0, 1.4 Hz, H-10), 9.26 (1H, dd*, J* = 9.0, 1.2 Hz, H-11), 4.13 (3H, s*,* 1-OCH_3_), 4.06 (3H, s*,* 2-OCH_3_). ^13^C NMR (CDCl_3_, 600 MHz) δ 145.3 (s, C-1), 158.2 (s, C-2), 108.3 (d, C-3), 157.5 (s, C-3a), 125.8 (s, C-4), 145.0 (d, C-5), 155.6 (s, C-6a), 123.3 (s, C-6b), 182.6 (s, C-7), 132.8 (s, C-7a), 129.3 (d, C-8), 129.8 (d, C-9), 135.7 (d, C-10), 129.7 (s, C-11), 135.8 (s, 11a), 120.0 (s, 11b), 60.1 (q, 1- OCH_3_), 56.4 (q, 1- OCH_3_). MS: m/z 291 (100%), 275 (15%), 248 (84%), 233 (9%), 188 (4%), 177 (12%). The molecular mass of **1** is *m/z* 291 amu which is consistent with the formula C_18_H_13_NO_3._ All the data for compound **1** were consistent with the reported values for lysicamine, which was first isolated from *Lysichiton camtschatcense* (Araceae) [28,29]. Lysicamine (**1**) has been widely isolated from several plant species [30] however; this is the first report on the presence of lysicamine (**1**) from *A*. *kummeriae* (Annonaceae).

Trivalvone (**2**): brown crystals (8.10 mg), m.p. 256-258°C), ^1^H NMR (CDCl_3_, 500 MHz) δ 6.87 (1H, s, H-3), 7.54 (1H, d, *J* = 4.1, H-4), 8.90 (1H, d, *J* = 4.1, H-5), 7.76 (1H, d, *J* = 9.0, 2.1, H-8), 7.35 (1H, t, *J* = 9.0, 2.1, H-9), 7.85 (1H, t, *J* = 9.0, 2.1, H-10), 10.20 (1H, d, *J* = 9.0, 2.1 H-11), 7.18 (1H, s, H-3´), 2.95 (2H, m, H-4´), 3.26 (2H, m, H-5´), 6.70 (1H, d, H-8´), 7.12 (1H, t, H-9´), 7.43 (1H, t, H-10´), 9.75 (1H, d, H-11´), 4.07 (3H, s, 2-OCH_3_), 4.01 (3H, s, 1´ -OCH_3_), 4.07 (3H, s, 2´ -OCH_3_), 2.15 (3H, s, 1´ - N-CH_3_). ^13^C NMR (CDCl_3_, 500 MHz) δ 181.0 (s, C-1), 151.3 (s, C-2), 107.5 (d, C-3), 127.9 (d, C-3a), 127.9 (d, C-4), 151.0 (d, C-5), 156.6 (s, C-6a), 122.6 (s, C-6b), 134.0 (s, C-7), 142.5 (s, C-7a), 132.6 (d, C-8), 128.7 (d, C-9), 127.0 (d, C-10), 121.9 (d, C-11), 122.7 (s, C-11a), 136.2 (s, C-11b), 145.9 (s, C-1´), 150.6 (s, C-2´), 112.8 (d, C-3´), 130.9 (s, C-3´ a), 25.6 (t, C-4´), 49.7 (t, C-5´ ), 143.8 (s, C-6´ a), 121.1 (s, C-6´ b), 122.4 (s, C-7´), 134.4 (s, C-7´ a), 127.9 (d, C-8´), 126.7 (d, C-9´), 126.6 (d, C-10´), 124.7 (d, C-11´), 126.2 (s, C-11´ a), 127.4 (s, C-11´ b), 56.3 (q, 2-OCH_3_), 60.0 (q, 1΄ -OCH_3_), 56.6 (q, 2΄ -OCH_3_), 41.6 (q, N-CH_3_). MS: m/z 554 ([M + 2]^+^, 90.4%), 553 ([M + 1]^+^, 41.3%), 292 (M/2 + H, 8.4%).

The molecular mass of **2** is *m/z* 552 amu, which is consistent with the formula C_36_H_28_N_2_O_4_. The absence of any fragmentation in the region *m/z* 552–292 suggested a dimeric structure for **2**, resulting from a C-7 → C-7´ oxidative coupling between the two aporphine units [31]. The NMR and MS data confirmed the structure of the bis-aporphine alkaloid, trivalvone (**2**)**,** a rare alkaloid first reported in 1990 from *Trivalvaria macrophylla* (Annonaceae) [31] and subsequently from *Piptostigma fugax* (Annonaceae) [32]. This is the first report on the presence of trivalvone (**2**) from *Annickia kummeriae* (Annonaceae).

Palmatine (**3**): yellow solid (1.52 g), m.p. 203–205°C, ^1^H NMR (CD_3_OD, 500 MHz) δ 7.63 (1H, s, H-1), 7.04 (1H, s, H-4), 3.30 (2H, t, *J* = 6.3, H-5), 4.95 (2H, t, *J* = 6.3, H-6), 9.75 (1H, br, s, H-8), 8.09 (1H, d, *J* = 9.1, H-11), 8.01 (1H, d, *J* = 9.1, H-12), 8.79 (1H, s, H-13), 3.94 (3H, s, 2-OCH_3_), 4.00 (3H, s, 3-OCH_3_), 4.22 (3H, s, 9-OCH_3_), 4.10 (3H, s, 10-OCH_3_). ^13^C NMR (CD_3_OD, 500 MHz) δ 110.4 (d, C-1), 151.3 (s, C-2), 154.2 (s, C-3), 112.7 (d, C-4), 130.4 (s, C-4a), 28.2 (t, C-5), 56.4 (t, C-6), 146.7 (d, C-8), 123.6 (s, C-8a), 146.1 (s, C-9), 152.3 (d, C-10), 128.4 (d, C-11), 124.9 (d, C-12), 135.6 (s, C-12a), 121.7 (d, C-13), 140.1 (s, C-13a), 120.8 (s, C-13b), 57.5 (q, 2-OCH_3_), 57.1 (q, 3-OCH_3_), 63.0 (q, 9-OCH_3_), 57.8 (q, 10-OCH_3_). MS: m/z 352 (75%), 337 (6%), 336 (7%), 308 (20%), 154 (100%), 77 (25%), 39 (20%). The mass spectrum of **3** exhibited a molecular ion peak at *m/z* 352, which is consistent with the formula C_21_H_22_NO_4_^+^ (D.B.E 11.5). The non-integer value of the index of hydrogen deficiency suggested that **3** could be a quaternary ammonium salt consistent with palmatine (**3**). All the observed data for **3** were consistent with the reported values for palmatine except for the interchange of H-11 and H-12 in ^1^H NMR [33,34]. Palmatine (**3**) has been previously reported from many plant families: Papaveraceae, Berberidaceae, Fumariaceae, Menispermaceae, Ranunculaceae, Rutaceae, Annonaceae, Magnoliaceae and Convolvulaceae [35].

Jatrorrhizine (**4**): orange crystals (40.82 mg), m.p. 204–206°C, ^1^H NMR (CDCl_3_, 600 MHz) δ 7.57 (1H, s, H-1), 6.76 (1H, s, H-4), 3.17 (2H, t, *J* = 6.1 Hz, H-5), 4.87 (2H, t, *J* = 6.1 Hz, H-6), 9.67 (1H, t, br, s, H-8), 8.07 (1H, d, *J* = 9.1 Hz, H-11), 7.96 (1H, d, *J* = 9.1 Hz, H-12), 8.68 (1H, s, H-13), 3.99 (3H, s, 2-OCH_3_), 4.19 (3H, s, 9-OCH_3_), 4.10 (3H, s, 10-OCH_3_). ^13^C NMR (CDCl_3_, 600 MHz) δ 109.6 (d, C-1), 150.9 (s, C-2), 152.1 (s, C-3), 116.6 (d, C-4), 130.5 (s, C-4a), 27.8 (t, C-5), 57.4 (t, C-6), 145.7 (d, C-8), 122.9 (s, C-8a), 145.9 (s, C-9), 151.5 (s, C-10), 128.1 (d, C-11), 124.2 (d, C-12), 135.7 (s, C12a), 120.2 (d, C-13), 141.1 (s, C-13a), 117.1 (s, C-13b), 56.7 (q, 2-OCH_3_), 62.4 (q, 9-OCH_3_), 57.7 (q, 10-OCH_3_). MS: m/z 338 (28%), 176 (55%), 154 (100%), 77 (29%), 41 (25%). MS exhibited a molecular ion peak at *m/z* 338 consistent with the formula C_20_H_20_NO_4_^+^ D.B.E of 11.5 indicating presence of a quaternary ammonium salt. All the data for compound **4** were consistent with the reported values for jatrorrhizine [35]. Jatrorrhizine (**4**) has been previously reported from several plant families: Papaveraceae, Berberidaceae, Fumariaceae, Menispermaceae, Ranunculaceae, Rutaceae, Annonaceae, Magnoliaceae and Convolvulaceae [35].

Columbamine (**5**): orange solid (34.2 mg, mp. 235–240°C), ^1^H NMR (CDCl_3_, 600 MHz) δ 7.51 (1H, s, H-1), 7.00 (1H, s, H-4), 3.24 (2H, t, *J* = 6.0 Hz, H-5), 4.92 (2H, t, *J* = 6.0 Hz, H-6), 9.74 (1H, t, br, s, H-8), 8.10 (1H, d, *J* = 9.0 Hz, H-11), 7.99 (1H, d, *J* = 9.0 Hz, H-12), 8.63 (1H, s, H-13), 3.95 (3H, s, 2-OCH_3_), 4.20 (3H, s, 9-OCH_3_), 4.10 (3H, s, 10-OCH_3_). ^13^C NMR (CDCl_3_, 600 MHz) δ 109.2 (d, C-1), 149.2 (s, C-2), 152.8 (s, C-3), 111.7 (d, C-4), 127.8 (s, C-4a), 27.7 (t, C-5), 57.4 (t, C-6), 146.1 (d, C-8), 123.2 (s, C-8a), 145.5 (s, C-9), 151.7 (s, C-10), 127.8 (d, C-11), 124.3 (d, C-12), 135.2 (s, C12a), 120.8 (d, C-13), 140.0 (s, C-13a), 120.5 (s, C-13b), 57.5 (q, 2-OCH_3_), 62.4 (q, 9-OCH_3_), 56.4 (q, 10-OCH_3_). MS: m/z 338 (28%), 176 (55%), 154 (100%), 77 (29%), 41 (25%). The MS of columbamine (**5**) exhibited a molecular ion peak at *m/z* 338 consistent with the formula C_20_H_20_NO_4_^+^ (D.B.E 11.5) confirming the presence of quaternary nitrogen atom. All the data were consistent with the reported values for columbamine (**5**) [35]. Columbamine (**5**) has been previously reported from several plant families: Papaveraceae, Berberidaceae, Fumariaceae, Menispermaceae, Ranunculaceae, Rutaceae, Annonaceae, Magnoliaceae and Convolvulaceae [35].

(−)-Tetrahydropalmatine (**6**): yellow amorphous solid (28.2 mg, m.p. 204–205°C), ^1^H NMR (600 MHz, CD_3_OD) δ 6.89 (1H, s, H-1), 6.90 (1H, s, H-4), 3.28 (1H, m, H-5_*eq*_), 3.33 (1H, m, H-5_*ax*_), 3.60 (1H, m, H-6_*eq*_), 3.84 (1H, m, H-6_*ax*_), 4.91 (1H, d, *J* = 15.7, H-8_*eq*_), 4.78 (1H, d, *J* = 15.7, H-8_*ax*_), 7.07 (1H, d, *J* = 8.5, H-11), 6.98 (1H, d, *J* = 8.5, H-12), 3.15 (1H, dd, *J* = 18, 10.3, H-13_*ax*_), 3.50 (1H, dd, *J* = 18, 5.7, H-13 _*eq*_), 4.76 (1H, dd, *J* = 10.3, 5.7, H-13a), 3.84 (3H, s, 2-OCH_3_), 3.85 (3H, s, 3-OCH_3_), 3.90 (3H, s, 9-OCH_3_), 3.87 (3H, s, 10-OCH_3_). ^13^C NMR (600 MHz, CD_3_OD) δ 111.3 (d, C-1), 151.6 (s, C-2), 150.4 (s, C-3), 115.5 (d, C-4), 125.7 (s, C-4a), 24.6 (t, C-5), 53.3 (t, C-6), 61.4 (d, C-8), 121.4 (s, C-8a), 147.1 (s, C-9), 153.1 (s, C-10), 115.0 (d, C-11), 125.0 (d, C-12), 123.7 (s, C12a), 35.4 (d, C-13), 67.7 (s, C-13a), 125.7 (s, C-13b), 53.5 (q, 2-OCH_3_), 56.4 (q, 3-OCH_3_), 63.1 (q, 9-OCH_3_), 56.1 (q, 10-OCH_3_). The MS of (−)-tetrahydropalmatine (**6**) exhibited molecular ion peak at *m/z* 356 consistent with the formulae C_21_H_27_NO_4_ (D.B.E 10). The odd molecular mass confirmed the presence of a neutral alkaloid. Comparison of the observed spectral data with literature values for (−)-tetrahydropalmatine (**6**) [35]. (−)-Tetrahydropalmatine (**6**) has been previously reported from several plant families: Papaveraceae, Berberidaceae, Fumariaceae, Menispermaceae, Ranunculaceae, Rutaceae, Annonaceae, Magnoliaceae and Convolvulaceae [35]. This is the first report on the presence of (−)-tetrahydropalmatine (**6**) from *Annickia kummeriae* (Annonaceae).

## Results and discussion

The *in vitro* anti-plasmodial, anti-trypanosomal, anti-leishmanial and lower cytotoxicity activity of extracts from *A*. *kummeriae* were previously published elsewhere [36]. Results of the fractionation of methanolic extract of *A*. *kummeriae* leaves by HSCCC are shown in Table 1. Of the 17 fractions 8 (47.1%) exhibited very strong anti-plasmodial activity against *P. falciparum* K1 strain (IC_50_ 0.05 ± 0.01-0.13 ± 0.02 μg/ml) with excellent selectivity (SI >692), 3 (17.6%) showed strong activity (IC_50_ 0.45 ± 0.15-0.87 ± 0.1 μg/ml) with satisfactory selectivity (SI 22.9-145.2) while the remaining 6 (35.3%) exhibited moderate activity (IC_50_ 1.0 ± 0.22-5.0 ± 0.31 μg/ml) with moderate selectivity (SI 18.0-90.0). The anti-plasmodial activity (IC_50_) and cytotoxicity (CC_50_) of the 17 HSCCC fractions were compared with the standard drugs: chloroquine, artemisinin and podophyllotoxin.

**Table 1:** **Anti-plasmodial activity (IC**_**50**_**) and cytotoxicity (CC**_**50**_**) of HSCCC fractions of *Annickia kummeriae *methanolic leaf extract**

**Fraction**	**Wt (mg)**	***P. falciparum *K1 IC**_**50 **_**(μg/ml)**	**Cytotoxicity CC**_**50 **_**(μg/ml)**	**SI**	**IC**_**50 **_**fr. IC**_**50 **_**CQ**	**IC**_**50 **_**fr. IC**_**50 **_**Art**	**IC**_**50 **_**fr. IC**_**50 **_**Pdx**
AKLM	15,000	0.12 ± 0.01	30.0 ± 0.8	250	1.9	60	3,333
AKLM1	562	5.0 ± 0.31	>90.0	>18	79.4	2500	>10,000
AKLM2	2,999	0.87 ± 0.1	20.0 ± 3.3	23	13.8	435	2,222
AKLM3	1,534	3.01 ± 0.81	78.0 ± 5.4	26	47.8	1,505	8,667
AKLM 4	157	1.34 ± 0.33	76.0 ± 1.44	57	21.3	670	8,444
AKLM 5	249	3.6 ± 0.2	>90.0	>25	57.1	1,800	>10,000
AKLM 6	427	0.45 ± 0.15	59.0 ± 1.5	131	7.1	225	6,556
AKLM 7	165	0.11 ± 0.02	>90.0	>818	1.7	55	>10,000
AKLM 8	221	0.09 ± 0.04	>90.0	>1,000	1.4	45	>10,000
AKLM 9	680	0.06 ± 0.02	>90.0	>1,500	1.0	30	>10,000
AKLM 10	1,295	0.05 ± 0.02	>90.0	>1,800	0.8	25	>10,000
AKLM 11	1,679	0.05 ± 0.01	>90.0	>1,800	0.8	25	>10,000
AKLM 12	1,056	0.06 ± 0.03	>90.0	>1,500	1.0	30	>10,000
AKLM 13	878	0.62 ± 0.4	>90.0	>145	9.8	310	>10,000
AKLM 14	948	1.0 ± 0.22	>90.0	>90	15.9	500	>10,000
AKLM 15	1,232	0.13 ± 0.02	>90.0	>692	2.1	65	>10,000
AKLM 16	416	0.09 ± 0.03	84.0 ± 3.91	933	1.4	45	9,333
AKLM 17	498	1.67 ± 0.43	>90.0	>54	26.5	835	>10,000

Fr. – HSCCC fraction of *Annickia kummeriae* leaves methanolic extract, *P. falciparum* K1 used for anti-plasmodial assays, rat myoblast L-6 cells used for cytotoxicity assays, *CQ* chloroquine (IC_50_ 0.063 ± 0.034 μg/ml), *Art* artemisinin (IC_50_ 0.002 ± 0.00001 μg/ml), *Pdx* podophyllotoxin (IC_50_ 0.009 ± 0.003 μg/ml).

Fractions AKLM 9–12 (IC_50_ 0.05 ± 0.01-0.09 ± 0.04 μg/ml, SI 1,000.0-1,800.0) were of particular interest since the anti-plasmodial activity compared very well to CQ, and was only 30-fold lower than that of artemisinin, and is not cytotoxic. Others with promising anti-plasmodial activity included: AKLM 8 (0.09 ± 0.004 μg/ml, SI >1,000, 1.4 and 45 fold lower than CQ and artemisinin, respectively), AKLM 16 (IC_50_ 0.09 ± 0.03 μg/ml, SI 933.3, 1.4 and 45 fold lower than CQ and artemisinin, respectively), AKLM 7 (IC_50_ 0.11 ± 0.02 μg/ml, SI >818.2; 1.7 and 55 fold lower than CQ and artemisinin, respectively), AKLM 15 (IC_50_ 0.13 ± 0.02 μg/ml, SI >692, 2.1 and 65 fold lower than CQ and artemisinin, respectively) and AKLM 6 (IC_50_ 0.45 ± 0.15 μg/ml, SI 131.1, 7.1 and 225 fold lower than CQ and artemisinin, respectively) all of which were not toxic. HSCCC re-fractionation of AKLM 2 (IC_50_ 0.87 ± 0.1 μg/ml, SI 22.9) gave 11 sub-fractions but only two (AKLM 2.10 and AKLM 2.11 with IC_50_ 0.64 ± 0.34 and 0.89 ± 0.20 μg/ml, respectively) showed moderate anti-plasmodial activity against *P. falciparum* K1 strain as the mother fraction: (Table 2).

**Table 2:** **Anti-plasmodial activity (IC**_**50**_**) and cytotoxicity (CC**_**50**_**) data of sub-fractions of fraction 2 of *Annickia kummeriae *methanolic leaf extract**

**Fraction**	**Weight (mg)**	***P. falciparum *K1 IC**_**50 **_**(μg/ml)**	**Cytotoxicity CC**_**50 **_**(μg/ml)**	**SI**	**IC**_**50 **_**fr. IC**_**50**_**CQ**	**IC**_**50 **_**fr. IC**_**50 **_**Art**	**CC**_**50 **_**fr. CC**_**50 **_**Pdx**
AKLM 2	2,500	0.90 ± 0.11	21.0 ± 3.87	23.3	14	450	3,500
AKLM 2.1	15.3	5.0 ± 1.31	>90	18.0	78	2,500	>15,000
AKLM 2.2	26.9	1.09 ± 0.26	79.1 ± 7.60	72.6	17	545	13,183
AKLM 2.3	57.6	4.11 ± 0.29	53.1 ± 9.20	12.9	64	2,055	8,850
AKLM 2.4	98.7	1.16 ± 0.18	13.8 ± 1.80	11.9	18	580	2,300
AKLM 2.5	126.1	3.63 ± 0.04	24.6 ± 3.30	6.8	57	1,815	4,100
AKLM 2.6	115.7	1.23 ± 0.31	39.5 ± 2.20	32.1	19	615	6,583
AKLM 2.7	239.0	3.41 ± 0.48	85.4 ± 4.60	25.0	53	1,705	14,233
AKLM 2.8	478.3	5.0 ± 0.53	>90	18.0	78	2,500	>15,000
AKLM 2.9	351.8	2.40 ± 0.57	56.7 ± 8.00	23.6	38	1,200	9,450
AKLM 2.10	301.9	0.89 ± 0.20	35.3 ± 5.31	39.7	14	445	5,883
AKLM 2.11	672.8	0.64 ± 0.34	44.7 ± 4.45	69.8	10	320	7,450

*HSCCC* High speed counter current chromatography; *AKLM Annickia kummeriae* leaf methanol extract, *P. falciparum* K1 used for anti-plasmodial assays, rat myoblast L-6 cells used for cytotoxicity assays, *CQ* chloroquine (IC_50_ 0.063 ± 0.034 μg/ml), Art - artemisinin (IC_50_ 0.002 ± 0.00001 μg/ml), Pdx - podophyllotoxin (IC_50_ 0.009 ± 0.003 μg/ml).

In fact the remaining 9 sub-fractions exhibited lower antiplasmodial activity (IC_50_ 1.09 ± 0.26-5.0 ± 0.53 μg/ml) and poor to satisfactory selectivity (SI 11.9-72.6) than the mother fraction. In the comparison of the antiplasmodial activity (IC_50_), HSCCC fraction AKLM 2.10 showed 13.9 and 445 fold lower activity than CQ and artemisinin, respectively) whereas, AKLM 2.11 revealed 10 and 320 fold lower activity than CQ and artemisinin, respectively). Both fractions were not cytotoxic. The methanolic leaf extract of *A. kummeriae* gave four pure alkaloids: lysicamine (**1**), trivalvone (**2**), palmatine (**3**), jatrorrhizine (**4**), and a pair of inseparable mixtures of two alkaloids each: jatrorrhizine (**4**)/columbamine (**5**), and palmatine (**3)**/(−)-tetrahydropalmatine (**6**), which were tested for anti-plasmodial, anti-trypanosomal, anti-leishmanial and cytotoxic activity.

Bioassay guided HSCCC fractionation of AKLM 2, using *P. falciparum* K1 strain, led to two major alkaloids lysicamine (**1**), an aporphine alkaloid and trivalvone (**2**), a bis-aporphine alkaloid as confirmed by spectral analysis. Lysicamine (**1**) has been widely isolated from several plant species [30]. However, this is the first report on its isolation from *A*. *kummeriae* (Annonaceae). Trivalvone (**2**) is a rare bis-aporphine alkaloid first reported in 1990 from *Trivalvaria macrophylla* (Annonaceae) [31] and subsequently from *Piptostigma fugax* (Annonaceae) [32]. This is also the first report on its presence in *A*. *kummeriae* (Annonaceae).

Similarly, bioassay-led HSCCC fractionation of the combined antiplasmodial fractions AKLM 7-AKLM 10, led to the isolation of two protoberberine alkaloids, which were confirmed by spectral analysis as palmatine (**3**) [10,33-35,37] and jatrorrhizine (**4**) [34,35,37]. Likewise, bioassay-directed HSCCC fractionation of the combined anti-plasmodial fractions AKLM 4-AKLM 6 yielded an inseparable mixture (1.2:1.0) of protoberberine alkaloids, which were confirmed as jatrorrhizine (**4**) [34,35] and columbamine (**5**) [35,38,39] by spectral analysis. Bioassay-informed HSCCC fractionation of the anti-plasmodial fraction AKLM 16 gave an inseparable mixture (1.1:1.0) of protoberberine alkaloids, which upon spectral analysis were confirmed a palmatine (**3**) and (−)-tetrahydropalmatine (**6**) [33-35,40,41]. This is the first report on the presence of columbamine (**5**) and (−)-tetrahydropalmatine (**6**) in *A*. *kummeriae*.

The four pure alkaloids, lysicamine (**1**), trivalvone (**2**), palmatine (**3**), jatrorrhizine (**4**) and the two sets of mixtures of jatrorrhizine (**4**) with columbamine (**5**) and palmatine (**3**) with (−)-tetrahydropalmatine (**6**) were found to exhibit *in vitro* anti-plasmodial activity against the multi-drug resistant *P. falciparum* K1 strain, anti-trypanosomal activity against the *T. b. rhodesiense* STIB 900 and anti-leishmanial activity against *L. donovani* axenic MHOM-ET-67/82 strain (Table 3).

**Table 3:** **Anti-protozoal activity (IC**_**50**_**) and cytotoxicity (CC**_**50**_**) data of alkaloids from *Annickia kummeriae***

	***P. falciparum *K1**		***T.b. rhodesiense***		***L. donovani***		**L-6 cells**
**Compound**	**IC**_**50 **_**±S.E (μg/ml)**	**SI**	**IC**_**50**_**±S.E (μg/ml)**	**SI**	**IC**_**50**_**±S.E (μg/ml)**	**SI**	**CC**_**50 **_**± S.E (μg/ml)**
Lysicamine (**1**)	2.4 ± 0.642	1.5	3.7 ± 0.001	2.3	2.7 ± 0.001	1.7	1.6 ± 0.01
Trivalvone (**2**)	1.6 ± 0.232	28.3	14.3 ± 0.001	3.2	2.9 ± 0.001	15.6	45.3 ± 0.02
Palmatine (**3**)	0.080 ± 0.001	1,154	3.2 ± 0.004	28.1	7.8 ± 0.001	11.5	>90
Jatrorrhizine (**4**)	0.24 ± 0.002	375.0	4.2 ± 0.002	21.4	20.4 ± 0.03	4.4	>90
Jatrorrhizine (**4**)/columbamine (**5**)	0.14 ± 0.017	358.6	4.0 ± 0.001	12.6	13.1 ± 0.02	3.8	50.2 ± 0.08
Palmatine (**3**)/tetrahydro- palmatine (**6**)	0.098 ± 0.002	629.6	4.3 ± 0.005	14.4	7.0 ± 0.06	8.81	61.7 ± 0.01

*P. falciparum –* K1 strain, *T. b. rhodesiense* – STIB 900 strain, *L. donovani -* MHOM-ET-67/L82*,* L-6 - rat skeletal myoblast cells, IC_50_ – inhibitory concentration for 50% of tested parasites, CC_50_ – cytotoxic concentration for 50% of tested cells, chloroquine IC_50_ 0.063 ± 0.03, artemisinin IC_50_ 0.002 ± 0.0001, melarsoprol IC_50_ 0.002 ± 0.0001, miltefosine IC_50_ 0.11 ± 0.001, podophyllotoxin IC_50_ 0.009 ± 0.0003.

Four protoberberine alkaloids showed strong *in vitro* activity against *P. falciparum* K1 strain (IC_50_ 0.08 ± 0.001-0.24 ± 0.002 μg/ml) singly and as mixtures and good selectivity (SI >375) while the remaining two aporphine alkaloids exhibited moderate anti-plasmodial activity (IC_50_ 1.6 ± 0.23-2.4 ± 0.04 μg/ml) singly and poor to moderate selectivity (SI 1.6–28.8). Palmatine (**3**) exhibited the strongest anti-plasmodial activity against *P. falciparum* K1 strain (IC_50_ 0.08 ± 0.001 μg/ml) and a good selectivity (SI 1,154). Jatrorrhizine (**4**) also showed strong anti-plasmodial activity (0.24 ± 0.002 μg/ml) and good selectisity (SI >375). Protoberberine alkaloids were of particular interest as they showed strong anti-plasmodial activity which was very close to that of chloroquine as shown in Table 4. Our data indicate that, palmatine (**3**) and jatrorrhizine (**4**) with other protoberberine alkaloids such as columbamine (**5**) and (−)-tetrahydropalmatine (**6**) are active constituents responsible for the antiplasmodial activity of *A. kummeriae.* However, the protoberberines and the monomeric aporphine alkaloids were only moderately active against *T. b. rhodesiense* STIB 900 strain *in vitro* (IC_50_ 2.8 ± 0.001–4.3 ± 0.0005 μg/ml) with moderate selectivity (SI 14.4-28.1) whereas the *bis*-aporphine alkaloid, trivalvone (**2**) was inactive (IC_50_ 14.3 ± 0.001 μg/ml). Similarly, the two aporphine alkaloids showed moderate activity against *L. donovani* MHOM-ET-67/L82 axenic amastigotes *in vitro*: lysicamine (**1**) (IC_50_ 2.7 ± 0.0001 μg/ml) with no selectivity (SI 1.5) and trivalvone (**2**) (IC_50_ 2.9 ± 0.0001 μg/ml) with moderate selectivity (SI 15.6) while the remaining four protoberberine alkaloids were inactive (IC_50_ 7.0 ± 0.001-20.4 ± 0.001 μg/ml). Moderate to mild anti-leishmanial activity (23.6-185.5 folds) was noted for all the isolated compounds compared to miltefosine (IC_50_ 0.11 ± 0.001 μg/ml) as shown in Table 4.

**Table 4:** **Comparison of anti-protozoal activity (IC**_**50**_**) and cytotoxicity (CC**_**50**_**) of alkaloids from *Annickia kummeriae *with standard drugs**

**Compound**	**IC**_**50 **_**cpd IC**_**50 **_**CQ**	**IC**_**50 **_**cpd IC**_**50 **_**Art**	**IC**_**50 **_**cpd IC**_**50 **_**Mel**	**IC**_**50 **_**cpd IC**_**50 **_**Milt**	**CC**_**50 **_**cpd CC**_**50 **_**Pdx**
Lysicamine (**1**)	38.1	1,200.0	1,850.0	23.6	177.8
Trivalvone (**2**)	25.4	800.0	7,150.0	26.4	5,033.3
Palmatine (**3**)	1.3	40.0	1,600.0	70.9	>10,000
Jatrorrhizine (**4**)	3.8	120	2,100.0	185.5	>10,000
Jatrorrhizine (**4**) + columbamine (**5**) (1.2:1.0)	2.2	69.0	2,000.0	119.1	5,577.8
Palmatine (**3**) + **(−)-**tetrahydropalmatine (**6**) (1.1:1.0)	1.6	49.0	2,150.0	63.64	6,855.6

*Cpd* isolated compound, *CQ* chloroquine, *Art* artemisinin, *Mel* melarsoprol, *Pdx* podophyllotoxin.

The literature indicate that plants that contain protoberberine and aporphine alkaloids are used in folkloric medicine as anti-hypertensive, anti-cancer, anti-septic, sedatives, analgesics, anti-inflammatory, anti-fungal, anti-bacterial and anti-protozoal [21,40]. The *in vitro* anti-plasmodial activity of protoberberine alkaloids has been previously reported. However, none of them has been shown to be active *in vivo*[16-19,35]. Oxygenation at C-2, C-3 (ring A) and C-9, C-10 (ring D) together with the presence of quaternary nitrogen atom in position 7 in protoberberine alkaloids have already been identified as the structural motifs required for strong antiplasmodial activity [42]. The relationship between the oxygenation and the antiplasmodial activity provides clues for possible molecular frameworks for synthesis and structure-activity relationship studies which might lead to the identification of pharmacophore(s) for new generation of isoquinoline anti-plasmodial drug(s).

## Conclusion

To the best of our knowledge, this is the first report on the anti-plasmodial and anti-leishmanial activity of *A. kummeriae*, *in vitro* anti-trypanosomal activity of palmatine (**3**); anti-plasmodial, anti-trypanosomal, anti-leishmanial and cytotoxicity activity of trivalvone (**2**); anti-leishmanial and anti-trypanosomal activity of jatrorrhizine (**4**) and of the two sets of mixtures: jatrorrhizine (**4**)/columbamine (**5**) (1.2:1.0) and palmatine (**3**)/(−)-tetrahydropalmatine (**6**) (1.1:1.0). The present phytochemical and pharmacological results indicate that *A. kummeriae*, a traditional remedy for malaria and fever, exhibits a wide array of biological activities, which could be attributed to the constituent aporphine and protoberberine alkaloids. The protoberberine alkaloids exhibit good antiprotozoal activity in vitro and comparably low cytotoxicity. In contrast, the activity and selectivity of aporphine alkaloids is moderate. Given the reported lack of *in vivo* activity of protoberberine alkaloids, further investigations should focus on a better understanding of their pharmacokinetic properties, and on possible improvements through synthetic modifications.

## Competing interests

The authors declare no competing interests.

## Authors’ contributions

HMM conceived the project. HMM, TW, MC, MOO, DH and PD performed the experiments. IN, SMS, AH, US, MH and RB supervised the work. All authors evaluated the results and revised the manuscript for publication. All the authors read and approved the final manuscript.

## Pre-publication history

The pre-publication history for this paper can be accessed here:

http://www.biomedcentral.com/1472-6882/13/48/prepub
